# Cellulase recycling in high-solids enzymatic hydrolysis of pretreated empty fruit bunches

**DOI:** 10.1186/s13068-019-1476-x

**Published:** 2019-06-06

**Authors:** Jae Kyun Kim, Jungwoo Yang, So Young Park, Ju-Hyun Yu, Kyoung Heon Kim

**Affiliations:** 1 Department of Biotechnology, Graduate School, Korea University, Seoul, 02841 South Korea; 2 Center for Bio-based Chemical, Green Chemistry and Engineering Division, Korea Research Institute of Chemical Technology, Daejeon, 34602 South Korea

**Keywords:** Cellulase recycling, Empty fruit bunches, Enzymatic hydrolysis, High-solids loading, Hydrothermal pretreatment, Lignocellulose

## Abstract

**Background:**

The lignocellulosic biomass feedstocks such as empty fruit bunches (EFBs) prove to be potential renewable resources owing to their abundance, low prices, and high carbohydrate contents. Generally, the conversion of lignocellulosic biomass into chemicals, fuels, and materials mainly includes pretreatment, enzymatic hydrolysis, fermentation, and recovery of final products. To increase the economic viability of such processes, the cost of cellulase production and enzymatic hydrolysis should be reduced. For this, recycling cellulase can be considered for reducing the saccharification cost of lignocellulose. In this study, cellulase recycling for high-solids enzymatic hydrolysis (i.e., 20%) was evaluated in saccharification of hydrothermally-pretreated EFBs.

**Results:**

High-solids (20%) enzymatic hydrolysis of hydrothermally-pretreated empty fruit bunches with 40 FPU of Cellic CTec3/g glucan was carried out for cellulase recycling. In the second round of hydrolysis using a recycled enzyme, only 19.3% of glucose yield was obtained. The most important limiting factors for cellulase recycling of this study were identified as the enzyme inhibition by glucose, the loss of enzyme activities, and the non-productive binding of enzymes to insoluble biomass solids. To overcome these limitations, PEG was added prior to the first-round hydrolysis to reduce non-productive enzyme binding, glucose was removed from the enzyme fraction to be reused in the second-round hydrolysis, and EFB solids from the first-round hydrolysis were used in the second-round hydrolysis. These three additional measures with cellulase recycling resulted in a 3.5 times higher glucose yield (i.e., 68.0%) at the second round than that of the control, the second-round hydrolysis with cellulase recycling but without these measures.

**Conclusions:**

Because of the high obstacles found in this  study in achieving high saccharification yields in the high-solids saccharification of high-lignin lignocellulose with cellulase recycling, effective measures for improving enzymatic saccharification yields need to be accompanied with cellulase recycling.

## Background

An increasing demand of fossil fuels and the recent environmental issues have promoted the development of bioproducts from renewable resources [1, 2]. Among renewable resources, lignocellulosic biomass feedstocks such as empty fruit bunches (EFBs) are attractive due to their abundances, low prices, and high carbohydrate contents [3]. The typical conversion process of lignocellulosic biomass into chemicals, fuels, and materials mainly includes pretreatment, enzymatic hydrolysis, fermentation, and recovery of final products [4]. To increase the feasibility of commercializing such processes on a large scale, it is necessary to improve the process economics, and the cost reduction in cellulase production and enzymatic hydrolysis can be one example [5, 6].

Several approaches have been adopted to reduce cellulase costs, including protein engineering of cellulase [7], optimization of cellulase formulations [8], addition of accessory agents to cellulase such as synergistic proteins [9] and surfactants [10], and cellulase recycling [11]. Among these, cellulase recycling is a simpler and more practical approach that reuses the cellulase recovered from the hydrolysate from the preceding process into fresh biomass. To date, significant reduction in cellulase usage with glucose yields of 60–80% has been demonstrated on a laboratory scale via cellulase recycling: for example, four consecutive recyclings of 20 filter paper units (FPU) of Celluclast 1.5 L/g glucan for 2% (w/v) loading organosolv pretreated lodgepole pine, thus resulting in saving 80 FPU enzyme but losing 9.1% glucose yield [12]; three consecutive recyclings of 15 FPU of Spezyme CP/g glucan at 5% (w/v) for ammonia pretreated corn stover, thus resulting in saving 45 FPU enzyme but losing 26.8% glucose yield [13]; and three consecutive recyclings of 20 FPU of fungal cellulase/g glucan for 2% alkali pretreated wheat straw, thus resulting in saving 60 FPU enzyme but losing 12.8% glucose yield [14]. Nevertheless, such studies on cellulase recycling were performed in low-solids enzymatic hydrolysis (i.e., < 15%, w/v). In addition, these studies commonly indicated that solids containing lower lignin (i.e., < 10%, w/w) were suitable for cellulase recycling [12, 14].

In this study, cellulase recycling for high-solids enzymatic hydrolysis (i.e., 20%) was evaluated through the saccharification of hydrothermally-pretreated EFBs containing 30.8% of lignin. First, the effects of cellulase and EFBs loadings on the glucose yield were tested for high-solids enzymatic hydrolysis of pretreated EFBs. Next, the highly relevant limiting factors hindering cellulase recycling under the present experimental conditions were identified. Eventually, additional process-oriented measures were developed to overcome the limitations of cellulase recycling, thereby increasing the efficiency of cellulase recycling for high-solids enzymatic hydrolysis.

## Results and discussion

### Hydrothermal pretreatment of EFBs

According to the composition analysis in this study, the unpretreated EFBs comprised 37.4% (w/w) of glucan, 23.9% of hemicellulose, and 21.2% of lignin (Table 1). Although the compositions of EFBs may vary depending on the breeds and cultivation conditions, they generally comprise 23–65% (w/w) of glucan, 20–33% of hemicellulose, and 14–30% of lignin [15].

**Table 1:** Compositions of EFBs before and after hydrothermal pretreatment

Biomass component	Composition^a^ (% of total insoluble solids, w/w)		Recovery yield^b^ (%, w/w)
	Unpretreated	Pretreated	
Glucan	37.4 ± 0.6	54.7 ± 1.2	98.0
Hemicellulose (xylan, arabinan, galactan, and mannan)	23.9 ± 0.4	12.0 ± 0.3	33.6
Acid-insoluble lignin	21.1 ± 0.3	30.7 ± 0.7	92.7
Acid-soluble lignin	0.1 ± 0.0	0.1 ± 0.0	
Acetyl group	5.9 ± 0.1	NM	–
Ash	4.2 ± 0.1	0.8 ± 0.0	–
Hot-water extractives	10.6 ± 0.4	NA	–
Ethanol extractives	4.8 ± 0.2	NA	–

*NM* not measured; *NA* not applicable

^a^Experimental data are expressed as mean ± standard deviation

^b^Based on the insoluble recovery after washing with water, 67.0% (w/w)

After hydrothermal pretreatment, the recovery yield of insoluble solids was 67.0% (w/w) of initially loaded solids, and the glucan, hemicellulose, and lignin content (w/w) were 54.7%, 12.0%, and 30.8%, respectively, in the insoluble solids of pretreated EFBs (Table 1). Thus, the recovery yields (w/w) of the biomass components, glucan, hemicellulose, and lignin were determined as 98.0%, 33.6%, and 92.7%, respectively. The loss of glucan after the pretreatment was negligible at the present hydrothermal pretreatment at 190 °C for 15 min. The recovery yield of lignin was considerably high (92.7%). In contrast, a significant loss of hemicellulose (66.4%) was observed after the pretreatment. The substantial loss of hemicellulose and the insignificant losses of cellulose and lignin, observed in this study, were considered to follow the typical characteristics of the hydrothermal pretreatment.

### Effects of cellulase and EFBs loadings on the glucose yield

**Fig. 1:**
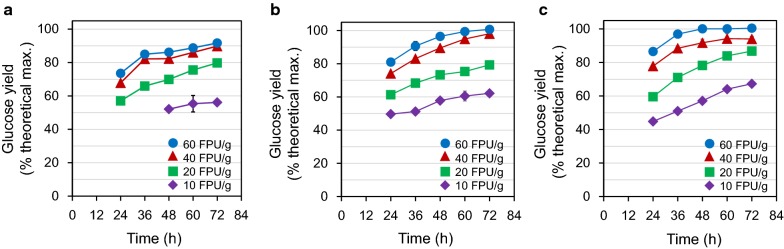
Enzymatic hydrolysis of hydrothermally-pretreated EFBs at the biomass loadings of **a** 30% (w/v), **b** 25%, and **c** 20%. Enzymatic hydrolysis was performed in 20 mL reaction mixtures with 10, 20, 40, and 60 FPU of Cellic CTec3/g glucan at pH 4.8, 50 °C, and 200 rpm. Samples were started being collected after the liquefaction occurred. Experimental data are expressed as mean ± standard deviation from two independent experiments

To investigate the effects of cellulase and EFB solids loadings on the glucose yields from the enzymatic saccharification of hydrothermally-pretreated EFBs, enzymatic hydrolysis was performed at 50 °C for 72 h by varying the loadings of EFB solids and cellulase (i.e., Cellic CTec3). In particular, the loading ranges of EFB solids and cellulase were 20–30% (w/v) and 10–60 FPU/g glucan, respectively. As a result, the glucose yield increased with increasing cellulase loading, but decreased with increasing solids loading (Fig. 1). The decrease in glucose yield with increasing a solids loading could be due to mass transfer limitation as previously reported [16].

At the 30% EFB solids loading (Fig. 1a), the liquefaction of solids slurry was slower in the initial period of enzymatic hydrolysis (e.g., 24 h) than at other EFB solids loadings. For instance, the liquid fraction of hydrolysates obtained with 10 FPU of Cellic CTec3/g glucan could not be collected even after 36 h (Fig. 1a). At higher solids loadings, mass transfer and mixing became limited due to the lack of free water, resulting in retarding the liquefaction of solids slurry [16]. Liquefaction is one of the key factors for efficient high-solids enzymatic hydrolysis, as it can affect the rheological behaviors between the biomass solids and endoglucanase [17]. Meanwhile, at the 30% EFB solids loading, the highest glucose yield, 91.7%, was obtained at a cellulase loading of 60 FPU/g glucan after 72 h. At the 25% EFB solids loading (Fig. 1b), liquefaction was faster than that at the 30% solids loading, and 79.3%, 98.1%, and 100.0% of glucose yields were obtained after 72 h at cellulase loadings of 20, 40, and 60 FPU/g glucan, respectively. At the 20% solids loading (Fig. 1c), 91.8% and 100.1% of glucose yields were obtained only after 48 h at cellulase loadings of 40 and 60 FPU/g glucan, respectively.

Among the cellulase loadings tested in Fig. 1, 10 FPU/g glucan is known as the most economically-feasible cellulase loading [18]. However, in this study, with 10 FPU/g glucan, only 56.1%, 62.2%, and 67.3% of glucose yields were obtained at 30%, 25%, and 20% solids loadings, respectively (Fig. 1), and these glucose yields were too low to readily observe the effect of cellulase recycling at high-solids loadings. Furthermore, with 20 FPU/g glucan, the glucose yield and volume of the liquid fraction separated for cellulase recycling were much lower than those with 40 FPU/g glucan at 48 h. Thus, 40 FPU and 60 FPU of Cellic CTec3/g glucan as the cellulase loadings, resulting in high glucose yield and titer, were selected to evaluate the feasibility of cellulase recycling at high-solids enzymatic hydrolysis of pretreated EFBs. In addition, based on the rate of enzymatic hydrolysis (Fig. 1a, b), 20% of EFB solids loading and 48 h time point, at which the glucose yield was saturated, were selected as the conditions for evaluating cellulase recycling. Under these saccharification conditions, 108.5 g/L and 118.4 g/L of glucose can be produced from 20% solids with 40 FPU and 60 FPU of Cellic CTec3/g glucan for 48 h, respectively.

### Recycling cellulase

**Fig. 2:**
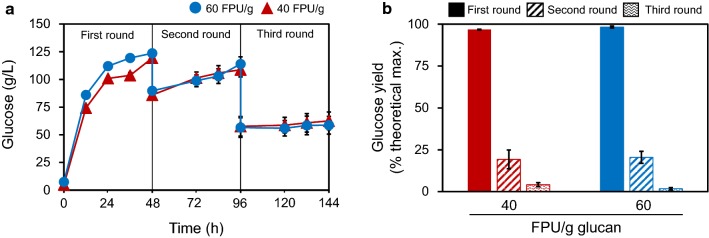
**a** Glucose titers and **b** glucose yields from the three rounds of enzymatic hydrolysis of hydrothermally-pretreated EFBs using recycled cellulase. Enzymatic hydrolysis was performed in 20 mL reaction mixtures at a 20% (w/v) solids loading with 40 and 60 FPU of Cellic CTec3/g glucan at pH 4.8, 50 °C, and 200 rpm. For cellulase recycling at the next-round enzymatic hydrolysis, the liquid fraction obtained from preceding rounds of enzymatic hydrolysis was directly applied to fresh pretreated EFBs with sodium citrate buffer (50 mM, pH 4.8). Experimental data are expressed as mean ± standard deviation from three independent experiments

To test the feasibility of cellulase recycling in the enzymatic hydrolysis of hydrothermally-pretreated EFBs, the liquid fractions of EFBs hydrolysates were collected after 48 h of the first-round enzymatic hydrolysis and were recycled further (i.e., the second and third rounds) for the enzymatic hydrolysis of pretreated EFBs. After the first-round enzymatic hydrolysis for 48 h, 96.7% and 98.3% of glucose yields were obtained with the glucose titers of 119.3 g/L and 123.6 g/L at the cellulase loadings of 40 FPU and 60 FPU/g glucan, respectively (Fig. 2a, b). At the end of the first-round enzymatic hydrolysis, the hydrolysate was filtered and 11.6 ± 0.6 mL of filtrate was obtained, which was 72.5% of the initial liquid volume of the hydrolysis reaction mixture. For the second-round enzymatic hydrolysis, the filtered liquid fraction was applied to the fresh pretreated EFBs. As a result, 19.3% and 20.5% of glucose yields were obtained during the second round of enzymatic hydrolysis at the cellulase loadings of 40 and 60 FPU/g glucan, respectively (Fig. 2b). For the third round of enzymatic hydrolysis, only 7.9 ± 0.4 mL of the liquid fraction filtrate was obtained due to the liquefaction in the second-round enzymatic hydrolysis. After the third-round enzymatic hydrolysis, glucose yields of less than 5% were obtained (Fig. 2b).

In this study, the increase in cellulase loading from 40 to 60 FPU/g glucan did not increase the efficiency of cellulase recycling, suggesting the existence of possible obstacles for cellulase recycling in the high-solids enzymatic hydrolysis, particularly the high lignin contents in biomass. Even at a low-solids enzymatic hydrolysis, cellulase recycling was hampered by the high lignin contents of biomass (i.e., 20–32%, w/w): 100% of glucose yield of steam-exploded loblolly pine at the first-round hydrolysis with 20 FPU Spezyme, 60% at the second round, and 15% at the third round [12]; 68% of glucose yield of acid-pretreated wheat straw (2%, w/w) at the first round with 20 FPU cellulase, 44% at the second round, and 27% at the third round [14]. Thus, the strategy of alleviating the binding of cellulase and lignin was suggested to overcome the limitations in the cellulase recycling [19].

### Limiting factors in cellulase recycling during high-solids enzymatic hydrolysis

#### Product inhibition of enzymes by glucose

**Fig. 3:**
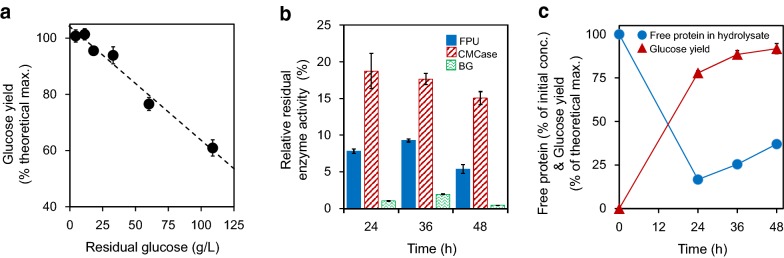
**a** Effect of residual glucose on the glucose yield of enzymatic hydrolysis of hydrothermally-pretreated EFBs. **b** Residual enzyme activities in the liquid fraction from the first-round enzymatic hydrolysis of pretreated EFBs. **c** Time course of the free protein concentration in the hydrolysate and the glucose yield (% of theoretical maximum) during the enzymatic hydrolysis of pretreated EFBs. Experimental data are expressed as mean ± standard deviation from two (enzymatic hydrolysis) or three independent experiments. *FPU* filter paper unit, *BG* β-glucosidase, *CMCase* carboxymethyl cellulase

Although high-solids enzymatic hydrolysis can produce high titer glucose, residual glucose during the enzymatic hydrolysis significantly limits the yield of enzymatic hydrolysis [20]. Considering the concentration of glucose to be as high as 85.0 g/L in the EFBs hydrolysates during commencement of the second-round enzymatic hydrolysis (Fig. 2a), such a high titer may seem to be a limiting factor for the high-solids enzymatic hydrolysis. Although the inhibition of cellulase by glucose is well known, the degree of glucose inhibition can be significantly affected by the types and concentrations of substrates and enzymes used [21]. Thus, to investigate whether the residual glucose is the limiting factor under the present experimental conditions, glucose was added at various amounts to the enzymatic hydrolysis of pretreated EFBs. As a result, as the residual glucose concentration increased from 0 to 108.0 g/L, the glucose yield decreased from 100.0 to 60.9% (Fig. 3a). This decreased glucose yield may be due to the product inhibition to cellulase by glucose [22]. Therefore, prior to the second-round enzymatic hydrolysis, 85.0 g/L, the initial glucose concentration, which was carried over from the filtrate of the liquid fraction of the first-round hydrolysate, needs to be reduced.

#### Loss of residual enzyme activity in the liquid fraction after enzymatic hydrolysis

At the typical enzymatic hydrolysis conditions for lignocellulose, pH 4.8 and temperature of 50 °C [23], β-glucosidase originating from *Trichoderma reesei* in Cellic CTec2 was found to bind onto lignin, thus resulting in a significant activity loss of residual β-glucosidase in the liquid fraction [24]. This loss of activity can lead to accumulation of cellobiose with producing less glucose [25]. In this study, the activity loss of β-glucosidase in Cellic CTec3 was hypothesized as one of the most relevant limiting factors in cellulase recycling, and the β-glucosidase activity was monitored in the first-round enzymatic hydrolysis of pretreated EFBs. In addition, the activities of total cellulase and CMCase (carboxymethyl cellulase) were also monitored. In particular, the endoglucanase, whose activity is expressed by CMCase, plays a key role in the liquefaction of high-solids enzymatic hydrolysis [17].

In this study, after the first-round enzymatic hydrolysis for 24 h, all of the three enzyme activities, namely, total cellulase, endoglucanase, and β-glucosidase, were retained less than 20% of their initial activities (Fig. 3b). Thus, it was supposed that productive binding of enzymes onto cellulose was dominant for 24 h, while non-productive binding of enzymes onto lignin was dominant after 48 h. In particular, β-glucosidase retained less than 1.1% of its initial activity after 24 h of enzymatic hydrolysis. Similar severe activity loss of β-glucosidase was reported in Cellic CTec2 when incubated with stream-pretreated EFBs comprising a high lignin content (e.g., 37.6%) [26], presumably due to the non-productive binding of β-glucosidase to lignin [24]. With regard to the total cellulase and CMCase activities, 5.4% and 15.0% were retained after the first-round enzymatic hydrolysis at 48 h (Fig. 3b). The higher loss of the total cellulase activity, which was measured by the glucose production from filter paper, than the endoglucanase activity may be due to the significant activity loss of β-glucosidase that primarily produces glucose from cellobiose. Accordingly, at the end of the first round of hydrolysis, cellobiose was not detected because the glucose yield reached almost 100%. However, at the end of the second round of hydrolysis, cellobiose accumulated up to 21.4 g/L (data not shown).

#### Binding of enzyme proteins to insoluble biomass solids

As observed in the non-productive binding of enzymes (e.g., β-glucosidase) to lignin [24], the significant losses of remaining enzyme activity in the liquid fraction in this study after enzymatic hydrolysis was hypothesized to be mainly due to non-productive binding of enzymes to the insoluble solids of pretreated EFBs including lignin [27]. To validate this hypothesis, protein adsorption during enzymatic hydrolysis was monitored (Fig. 3c). As a result, only 16.7% of the initial amount of proteins were recovered after 24 h of enzymatic hydrolysis. After the completion of enzymatic hydrolysis at 48 h, the amount of proteins recovered from the liquid fraction were still as low as 37% of its initial amount (Fig. 3c). These results indicated that most of enzyme proteins of Cellic CTec3 still remained in the solid fraction after enzymatic hydrolysis. Typically, enzymes bind onto solids immediately in the beginning of hydrolysis. Once enzymatic hydrolysis occurs, enzymes bound to solids start being detached from the solids [28]. However, approximately 40% of the initial enzymes still remain in the solids after the completion of enzymatic hydrolysis even in low-solids enzymatic hydrolysis [12, 28]. Considering that most of cellulose and hemicellulose were hydrolyzed after 48 h in this study, enzymes may have been adsorbed onto lignin. Thus, it was supposed that productive binding of cellulases onto cellulose was dominant during enzymatic hydrolysis according to the residual activity of cellulases in the liquid fraction. However, it was supposed that non-productive binding of cellulases onto lignin was dominant after the completion of enzymatic hydrolysis due to the depletion of cellulose.

### Overcoming the limiting factors in cellulase recycling

#### Effect of glucose separation on cellulase recycling

**Fig. 4:**
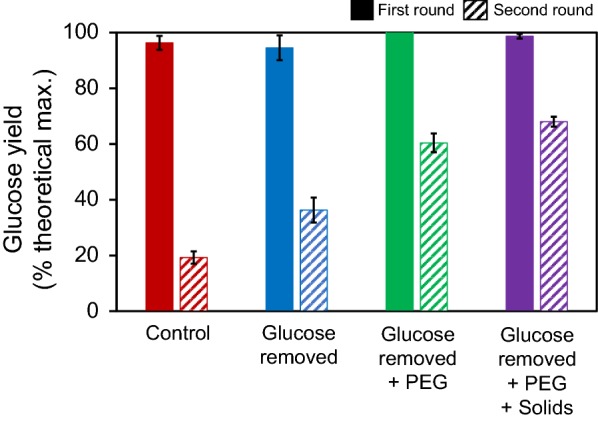
Effects of the glucose removal from the enzyme fraction to be recycled, the addition of PEG to reduce enzyme binding, and the use of biomass solids from the first-round enzymatic hydrolysis, on the glucose yield of the second round in the two-round enzymatic hydrolysis of hydrothermally-pretreated EFBs with cellulase recycling

To alleviate the product inhibition of enzymes by glucose, as observed earlier in this study, it is necessary to remove glucose from the liquid fraction of EFBs hydrolysates between the first- and second-round enzymatic hydrolysis. To accomplish this, the glucose and enzymes in the liquid fraction from the first-round enzymatic hydrolysis were separated using 10 kDa ultrafiltration (UF) membrane, and the UF filtrate containing glucose was separated from the cellulase-containing fraction. When switching from the liquid fraction without using UF to the UF-separated enzyme fraction for the second-round enzymatic hydrolysis, the glucose yield increased by 1.9 times, from 19.3 to 36.3% (Fig. 4).

#### Effect of polyethylene glycol on cellulase recycling

To alleviate the significant loss of β-glucosidase activity, presumably due to its non-productive binding of β-glucosidase onto lignin, as observed in this and other studies [24, 27], 62.5 mg of polyethylene glycol (PEG) 8000/g glucan was added prior to the first round of enzymatic hydrolysis of pretreated EFBs, as previously described [29]. In this study, when PEG was added, the glucose yield from the second-round enzymatic hydrolysis was 60.4%, which was 3.1 times higher than that of the untreated control (i.e., 19.3%; Fig. 4) and which was 1.6 times higher than that obtained after glucose separation. Therefore, not only the PEG addition but also the glucose separation was found to be effective in increasing the enzymatic hydrolysis of pretreated EFBs using recycled cellulases. This effect of PEG on the enzymatic hydrolysis was previously suggested to be due to the reduction of non-productive binding of cellulases onto insoluble solids of pretreated lignocellulose [29, 30].

#### Effect of used biomass solids with bound enzymes on cellulase recycling

So far in this study, to overcome the limiting factors of cellulase recycling, the separation of glucose and the addition of PEG were additionally performed and resulted in a 3.1 times yield increase in the second round of enzymatic hydrolysis compared to the glucose yield of the second round of cellulase recycling without these two measures. To furthermore increase the reusability of enzymes bound to the residual solids in the first-round enzymatic hydrolysis, 33.0% of the used solids from the first-round hydrolysis were directly applied to the second-round enzymatic hydrolysis. Meanwhile, when performing the enzymatic hydrolysis with more than 33.3% of used solids (2.6 g of 7.9 g), the liquefaction was rarely observed due to high viscosity at a high solids loading. After the second-round enzymatic hydrolysis, a glucose yield of 68.0% was obtained, which was 7.6% higher than that obtained after glucose separation and PEG addition, but without the reuse of the used solid fraction (Fig. 4). Overall, when introducing the additional three measures, glucose separation, PEG addition, and the reuse of the used solids fraction into the second-round enzymatic hydrolysis, the glucose yield increased by 3.5 times compared to that of control without these efforts.

Using the above three additional measures for cellulase recycle, when the three scenarios of enzymatic hydrolysis of pretreated EFBs, the two separate hydrolysis using 20 FPU of Cellic CTec3/g glucan, and the consecutive hydrolysis through cellulase recycling using 40 FPU/g glucan with or without PEG and used biomass solids, were compared for their overall glucose yield from the two rounds (Table 2). The overall glucose yield with cellulase recycling without the three measures was rather lower than that of the separate hydrolysis without cellulase recycle (Table 2) due to the low glucose yield in the second round. However, the cellulase recycling with the three additional measures showed a 6.6% higher glucose yield than that from the separate hydrolysis. These results imply that the cellulase recycling itself cannot benefit the overall glucose yield without additional measures for improving hydrolysis efficiency. Also, to further increase the glucose yield from the aspect of enzymes, the protein engineering and formulation of enzymes can be performed to reduce electrostatic interactions between enzymes and lignin and to achieve a more favorable distribution of enzymes during enzymatic hydrolysis.

**Table 2:** Effect of cellulase recycling on the overall glucose yields and total amounts of produced glucose (mean ± standard deviation) from the enzymatic hydrolysis of hydrothermally-pretreated EFBs

Mode of the two-round enzymatic hydrolysis	Hydrolysis round	Cellulase loading (FPU/g glucan)	Glucose yield (% of theoretical max.)	Total amount of glucose produced (g)^c^
Separate hydrolysis without cellulase recycling	R1^a^	20	78.2 ± 0.1	3.70 ± 0.00
	R2^b^	20	78.2 ± 0.1	
Cellulase recycling	R1^a^	40	98.7 ± 0.8	2.79 ± 0.15
	R2^b^	0	19.3 ± 5.7	
Cellulase recycling with the additional treatments	R1^a^	40	98.7 ± 0.8	3.94 ± 0.06
	R2^b^	0	68.0 ± 1.8	

^a^The first round of enzymatic hydrolysis

^b^The second round of enzymatic hydrolysis

^c^The overall glucose produced from 4 g of pretreated EFBs at the first round and 4 g of pretreated EFBs at the second round

## Conclusions

Cellulase recycling for high-solids enzymatic hydrolysis is important and challenging, especially, when pretreated biomass contains a high lignin content. In this study, it was found that cellulase recycling in the enzymatic  hydrolysis of hydrothermally-pretreated EFBs (20%, w/v) was hampered by product inhibition of enzymes by glucose, activity losses of enzymes after hydrolysis, and binding of enzymes to insoluble solids of biomass. To overcome these obstacles for cellulase recycling, glucose was removed from the enzyme fraction to be reused, PEG was added to reduce enzyme binding, and used EFB solids from the first-round hydrolysis were added to reuse enzymes bound to the solids. As a result, a 3.5 times higher glucose yield than that of untreated control was obtained.

## Methods

### Hydrothermal pretreatment of EFBs

The EFBs used in this study were provided by the Korindo Group (Jakarta, Indonesia). These EFBs were ground and sieved to obtain approximately 850 μm particle sizes, which were then subjected to hydrothermal pretreatment at 190 °C for 15 min at a solid-to-liquid ratio of 1:11.5 (w/v). Briefly, 800 g of dry weight EFBs were soaked in 16 L of deionized water at 50 °C for 16 h, and were dewatered by centrifugation. Thereafter, they were transferred into a 15 L pretreatment reactor (Hanwoul Engineering, Gunpo, Korea) containing 9.2 L of deionized water. The temperature of the pretreatment reactor was increased to 190 °C with a 50 min ramping time and was maintained at 190 °C for 15 min to pretreat the EFBs, which were then discharged into a fabric bag with approximately 6 μm diameter pores and washed with water. Eventually, the pretreated EFBs were dried in a freeze dryer (IlShinBioBase, Dongducheon, Korea) and stored at − 20 °C until further use.

### Compositional analysis of unpretreated and pretreated EFBs

Compositions of unpretreated and pretreated EFBs were analyzed according to the Laboratory Analytical Procedure (LAP) of National Renewable Energy Laboratory (NREL; Golden, CO) [31–33]. After the two-step acid hydrolysis, the sugar contents in the liquid fraction were measured using high-performance liquid chromatography (HPLC; Agilent 1100, Agilent Technologies, Waldronn, Germany) equipped with Aminex HPX-87H column (Bio-Rad, Hercules, CA). The acid-soluble lignin content in the liquid fraction was measured at 205 nm using a spectrophotometer (xMark, Bio-Rad, Hercules, CA). For determining the acid-insoluble lignin content, the liquid fraction was filtered and incubated in a furnace at 575 °C for 3 h, and the acid-insoluble lignin was weighed and expressed as the percentage (w/w) of the total dry weight of insoluble solids of biomass. To determine the ash content, EFBs were incubated at 575 °C for 24 h, and the weight of the residual ash was expressed as the percentage of the total biomass [31]. The recovery yield (%) of each component of insoluble biomass after pretreatment was determined by comparing the total dry weight of recovered solids and biomass composition before and after pretreatment (Table 1).

### Enzymatic hydrolysis and cellulase recycling

Enzymatic hydrolysis was performed in duplicate according to the LAP of NREL [23]. Briefly, for each enzymatic hydrolysis, pretreated EFBs with dry wt. of 4 g were transferred into a glass vial containing 20 mL of the total reaction mixture comprising sodium citrate buffer (50 mM, pH 4.8), 0.02% (w/v) of sodium azide, and 10–60 FPU of cellulase/g glucan. Thereafter, the mixture was incubated at 50 °C with shaking at 200 rpm for 72 h.

To examine cellulase inhibition by its product, enzymatic hydrolysis was performed in the presence of glucose at different concentrations, 6.3, 12.5, 25, 50, and 100 g/L. Glucose released during the enzymatic hydrolysis was measured using the HPLC, and the glucose yield (%) was expressed as the percentage of the theoretical maximum glucose to be released from the total amount of glucan in input pretreated EFBs. Cellulase used in this study was Cellic CTec3, which was provided by Novozymes Korea (Seoul, Korea), and the filter paper unit of Cellic CTec3 was measured at 191.6 FPU/mL.

For cellulase recycling, following enzymatic hydrolysis for 48 h, the insoluble solids and the liquid fraction of the hydrolysates were separated by filtration using a 0.2 μm polyethersulfone membrane filter (Thermo Fisher Scientific, Waltham, MA). The collected liquid fraction was mixed with 4 g of fresh EFBs and sodium citrate buffer (50 mM, pH 4.8), resulting in a 20% (w/v) solids loading in a 20 mL reaction mixture. Thereafter, the reaction mixture was incubated at 50 °C with shaking at 200 rpm for 48 h, and the glucose yield (%) was determined as described above. To determine the glucose released during the second- or third-round enzymatic hydrolysis, the carried glucose from the previous round was subtracted.

### Analysis of residual enzyme activity and protein concentration

After enzymatic hydrolysis for 48 h, the residual sugar from the liquid fraction of hydrolysate was completely removed using a dialysis membrane with a molecular cut-off of 10 kDa (SnakeSkin, Thermo Fisher Scientific, Waltham, MA) in 5 L of sodium citrate buffer (50 mM, pH 4.8) at 4 °C for 4 h. Thereafter, the enzymatic activity and protein concentration of the liquid fraction were measured.

The total cellulase activity in an enzymatic reaction mixture expressed as FPU/mL was determined according to the LAP of NREL [34]. Briefly, 0.5 mL of a diluted sample containing enzymes was incubated with 50 mg of filter paper strip (Whatman No. 1, Whatman, Maidstone, UK) and 1.0 mL of sodium citrate buffer at 50 °C for 60 min. The enzymatic reaction was terminated by adding 3.0 mL of a 3,5-dinitrosalicylic acid (DNS) agent at 95 °C for 5 min. Thereafter, the amount of released glucose was measured as reducing sugar at 540 nm using a microplate spectrophotometer (xMark). One unit of cellulase (FPU/mL) was defined as the amount of enzyme releasing 2.0 mg of glucose for 60 min.

The residual carboxymethyl cellulase (CMCase) activity in an enzymatic reaction mixture was determined by incubating 0.5 mL of a diluted sample containing enzymes with 0.5 mL of 2% (w/v) carboxymethyl cellulose (CMC; Sigma-Aldrich, St. Louis, MO) at 50 °C for 30 min [35]. The enzymatic reaction was terminated by adding a DNS agent, and the amount of released glucose was measured as reducing sugar at 540 nm. One unit of CMCase was defined as the amount of enzyme releasing 0.5 mg of glucose for 30 min.

The residual β-glucosidase (BG) activity in an enzymatic reaction mixture was determined, as previously described [36]. Briefly, 0.5 mL of a diluted sample containing enzymes was incubated with 1.0 mL of 2 mM of *p*-nitrophenyl-β-d-glucopyranoside (*p*NPG; Carl Roth, Karlsruhe, Germany) at 50 °C for 10 min. The enzymatic reaction was terminated by adding 2.0 mL of 1 M sodium carbonate. The amount of *p*-nitrophenol released from *p*NPG during the enzymatic reaction was measured at 410 nm. One enzyme unit of β-glucosidase was defined as the amount of enzyme releasing 1 μmol of *p*-nitrophenol per min. The protein concentration was measured using a bicinchoninic acid protein kit (Thermo Scientific, Rockford, IL).

## Data Availability

The datasets used and/or analyzed during the current study are available from the corresponding author on reasonable request.

## Abbreviations

EFBs: empty fruit bunches
FPU: filter paper unit
CMC: carboxymethyl cellulose
CMCase: carboxymethyl cellulase
UF: ultrafiltration
PEG: polyethylene glycol
HPLC: high-performance liquid chromatography
DNS: dinitrosalicylic acid
BG: β-glucosidase
*p*NPG: *p*-nitrophenyl-β-d-glucopyranoside
